# Theoretical and experimental study on the electronic and optical properties of K_0.5_Rb_0.5_Pb_2_Br_5_: a promising laser host material

**DOI:** 10.1039/d0ra00718h

**Published:** 2020-03-17

**Authors:** Tuan V. Vu, A. A. Lavrentyev, B. V. Gabrelian, Dat D. Vo, Hien D. Tong, N. M. Denysyuk, L. I. Isaenko, A. Y. Tarasova, O. Y. Khyzhun

**Affiliations:** Division of Computational Physics, Institute for Computational Science, Ton Duc Thang University Ho Chi Minh City Vietnam vuvantuan@tdtu.edu.vn voduydat@tdtu.edu.vn; Faculty of Electrical & Electronics Engineering, Ton Duc Thang University Ho Chi Minh City Vietnam; Department of Electrical Engineering and Electronics, Don State Technical University 1 Gagarin Square 344010 Rostov-on-Don Russian Federation; Department of Computational Technique and Automated System Software, Don State Technical University 1 Gagarin Square 344010 Rostov-on-Don Russian Federation; Faculty of Engineering, Vietnamese German University Binh Duong Vietnam; Frantsevych Institute for Problems of Materials Science, National Academy of Sciences of Ukraine 3 Krzhyzhanivsky Street 03142 Kyiv Ukraine khyzhun@ipms.kiev.ua; Novosibirsk State University 630090 Novosibirsk Russian Federation; V. S. Sobolev Institute of Geology and Mineralogy, SB RAS 630090 Novosibirsk Russian Federation

## Abstract

The data on the electronic structure and optical properties of bromide K_0.5_Rb_0.5_Pb_2_Br_5_ achieved by first-principle calculations and verified by X-ray spectroscopy measurements are reported. The kinetic energy, the Coulomb potential induced by the exchange hole, spin-orbital effects, and Coulomb repulsion were taken into account by applying the Tran and Blaha modified Becke–Johnson function (TB-mBJ), Hubbard U parameter, and spin-orbital coupling effect (SOC) in the TB-mBJ + U + SOC technique. The band gap was for the first time defined to be 3.23 eV. The partial density of state (PDOS) curves of K_0.5_Rb_0.5_Pb_2_Br_5_ agree well with XES K Ll and Br Kβ_2_, and XPS spectra. The valence band (VB) is characterized by the Pb-5d_3/2_ and Pb-5d_5/2_ sub-states locating in the vicinities of −20 eV and −18 eV, respectively. The VB middle part is mainly formed by K-3p, Rb-4p and Br-4s states, in which the separation of Rb-4p_3/2_ and Rb-4p_1/2_ was also observed. The strong hybridization of Br-p and Pb-s/p states near −6.5 eV reveals a major covalent part in the Br–Pb bonding. With a large band gap of 3.23 eV, and the remarkably high possibility of inter-band transition in energy ranges of 4–7 eV, and 10–12 eV, the bromide K_0.5_Rb_0.5_Pb_2_Br_5_ is expected to be a very promising active host material for core valence luminescence and mid-infrared rare-earth doped laser materials. The anisotropy of optical properties in K_0.5_Rb_0.5_Pb_2_Br_5_ is not significant, and it occurs at the extrema in the optical spectra. The absorption coefficient *α*(*ω*) is in the order of magnitude of 10^6^ cm^−1^ for an energy range of 5–25 eV.

## Abstract 2

The data on the electronic structure and optical properties of bromide K_0.5_Rb_0.5_Pb_2_Br_5_ achieved by first-principle calculations and verified by X-ray spectroscopy measurements are reported.

## Graphical abstract

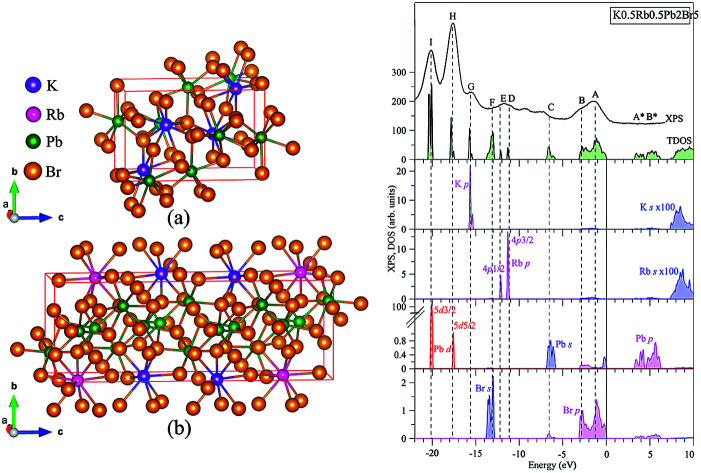

## 1 Introduction

Laser materials have been studied for many years due to their applications in many areas requiring high technologies such as global communication, medicine, diagnostic analysis, sensors, security, and defense.^1–3^ As a result of this long process of research and development, a variety of laser materials has been discovered which can be divided into solid, liquid, gas, and plasma groups.^4^ Among these, the solid-state lasers appear to be more advantageous for commercial manufacturing and practical usages where the compactness and simplicity of maintenance are required. For the last two decades, rare earth-doped borates and halides have been intensively studied and developed as they possess outstanding properties including good physical and chemical stability, high optical quality, large dimension, and moisture resistance.^5,6^ Recently, increasing attention has been paid to the rare-earth doped double halides as they can operate in the mid-IR range.^7^ Especially, the active crystal hosts with general formula APb_2_B_5_ (A = K, Rb; and B = Cl, Br) are of great interest owing to their low phonon energy of about 140 cm^−1^ which inhibits the non-radiative transition rate and increases the quantum yield.^8–13^ Rubidium- and potassium-bearing lead bromides, RbPb_2_Br_5_ (RPB) and KPb_2_Br_5_ (KPB), can be considered representative laser materials in this group because they are non-hygroscopic and they can readily incorporate with rare-earth (RE) ions.^12–17^ Thus, the composition of these laser materials can be varied for a wider range of applications. However, there are still challenges to be resolved before applying these materials in practical uses. The KPB compounds are reported to be thermal unstable,^15,18,19^ in which the phase-transition causes isotropy breaking to form light scattering centers. Thus, the emission generation is reduced due to optical losses. Meanwhile, the RPB compounds undergo no phase -transition even at melting point.^19^ The drawback of RPB compounds is that RE ion concentration in the crystals is an order of magnitude lower than in KPB crystals. To combine the positive properties of both compounds, KPB and RPB, many experiments have been made to define the best value of *x* in K_*x*_Rb_1−*x*_Pb_2_Br_5_ materials.^20–22^ It was experimentally found that the K_0.5_Rb_0.5_Pb_2_Br_5_ compound contains sufficient RE ion concentration for a high emission rate. At the same time, the phase-transition of this material occurs only at the melting point.^22^ This result indicates that K_0.5_Rb_0.5_Pb_2_Br_5_ appears to be a very promising laser material which is suitable for commercial manufacturing and practical uses. For this purpose, the XPS spectrum of K_0.5_Rb_0.5_Pb_2_Br_5_ was reported,^22^ and its thermal expansion was also studied.^23^

To our knowledge, there is no theoretical study on the electronic structure of K_0.5_Rb_0.5_Pb_2_Br_5_, which is, in fact, very necessary for understanding the inter-band transition of optical electrons in the process of amplification and generation of radiation, or the effect of valence electrons on phonon energy *via* electron–phonon coupling.^24^ Among the optical properties of K_0.5_Rb_0.5_Pb_2_Br_5_ only the absorption edge was reported.^22^ Although the absorption edge of RPB compounds is observed to depend on the polarization,^13,22^ it has not been studied for the case of K_0.5_Rb_0.5_Pb_2_Br_5_. It is worth mentioning that the polarization of optical properties, and photoelectron distribution can influence the cross section, and laser–material interaction, meanwhile the laser writing is affected by light polarization.^25,26^ This quaternary bromide was not studied also employing the method of X-ray emission spectroscopy (XES) providing information on the partial densities of states of a solid. To overcome the above drawbacks, in this paper, a theoretical study on the electronic and optical properties of K_0.5_Rb_0.5_Pb_2_Br_5_ is presented.

Recently, the density functional theory (DFT) has been successfully performed to study many systems including monolayers, nanoribbons, and bulks.^27–32^ The DFT calculations can be used to not only validate experimental results but also to give insight to the electronic, and optical properties of a semiconductor.^27^ In current study, the DFT calculations were performed by applying augmented plane-wave method (APW)^33,34^ in the framework of Kohn–Sham equations.^35^ Because K_0.5_Rb_0.5_Pb_2_Br_5_ is a semiconductor with excited processes, both generalized gradient approximation of Perdew, Burke, and Ernzerhof (GGA-PBE)^36^ and Tran and Blaha modified Becke–Johnson (TB-mBJ) functional^37,38^ were used to simulate the exchange–correlation interaction to evaluate the effect of kinetic energy as well as exchange interaction of exciting hole on the electronic structure. The spin-orbital coupling (SOC) effect,^39^ and the Hubbard parameter U^40,41^ were also employed in the calculations as they play a very important role in a system with heavy elements likes Pb.^42–51^

## 2 Computational details

**Fig. 1:**
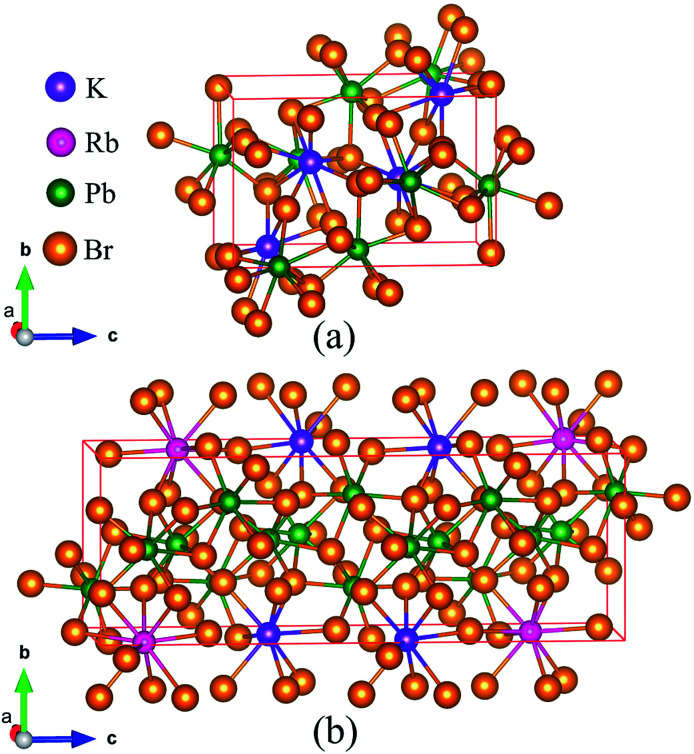
(a) KPb_2_Br_5_ crystal structure and (b) supercell of 1 × 1 × 2 used for optimization of K_0.5_Rb_0.5_Pb_2_Br_5_ structure.

As the crystal structure of K_0.5_Rb_0.5_Pb_2_Br_5_ has not been available in any published studies, the simulation was based on the monoclinic structure of KPb_2_Br_5_, as shown in Fig. 1(a), with space group *P*2_1_/*c* and lattice parameters *a* = 9.314 Å, *b* = 8.412 Å, *c* = 13.053 Å and *β* = 90.097°.^22^ The equilibrium structure of K_0.5_Rb_0.5_Pb_2_Br_5_ was obtained by optimizing a 1 × 1 × 2 supercell of KPb_2_Br_5_ structure, in which one K atom was replaced by one Rb atom as shown in Fig. 1(b).

The optimized structure of K_0.5_Rb_0.5_Pb_2_Br_5_ was used for calculating the electronic properties, in which the density functional theory as implemented in WIEN2k codes^52^ was used. The full potential of the current system was simulated by applying Muffin-tin (MT) model and the augmented plane wave plus local-orbital (APW + lo) method,^34,53^ where the potential does not depend on shape-approximation. The Muffin-tin sphere radius (*R*_MT_) of 2.5 a.u. was used for K, Rb, Pb and Br elements. The Fourier series were applied to expand the charge density with *G*_max_ = 14 (a.u.)^−1^, while the wavefunctions were expanded within Brillouin zone involving 1000 *k*-points with the maximum angular momentum *l*_max_ = 10, and *R*^MT^_min_*k*_max_ = 8. The exchange–correlation interaction was modeled by GGA-PBE^36^ and TB-mBJ^37,38^ methods with and without SOC^39^ and Hubbard U parameters. The Hubbard U parameter of 0.43 Ry was chosen for 5d states of Pb, 3p states of K and 4p states of Rb because such an approach was proven to give reliable theoretical results.^40,41^ The self-consistent process was converged when the difference in charge density is less than 10^−4^ Ry.

## 3 Experimental

In the present XES experiments, we use the optical quality K_0.5_Rb_0.5_Pb_2_Br_5_ crystal synthesized as described in detail in ref. 22 and cut at the (001) plane. Its XPS spectrum of valence electrons was measured as reported elsewhere.^22^ The X-ray emission Br Kβ_2_ spectrum (M_II,III_ → K transition) yielding information on the energy distribution of bromine 4p electronic states was measured using a DRS-2M spectrograph equipped with a quartz crystal as dispersion element (the (0001) reflecting plane bent according to Johann technique) and the spectrum was excited employing a BKhV-7 X-ray tube (Au anode). The spectrograph resolution, Δ*E*_min_, in the energy range corresponding to the region which covers the Br Kβ_2_ spectrum measured in the third reflection order was better than 0.25 eV and the operation regimes of the BKhV-7 X-ray tube were follows: accelerating voltage 45.5 kV and anode current 69.0 mA. The XES K Ll spectrum arising due to the L_III_ → M_I_ transition and representing information on the energy distribution of potassium 4s electronic states was acquired in the second order of reflection with spectrometer energy resolution better than 0.3 eV using an RSM-500 spectrometer supplied with a diffraction grating (600 groves per 1 mm and curvature radius equal to 6026 mm). Operation regimes of an electron gun when acquiring the XES K Ll spectrum were the following: accelerating voltage 5.3 kV and anode current 5.7 mA.

## 4 Results and discussion

### 4.1 Electronic structure of K_0.5_Rb_0.5_Pb_2_Br_5_

**Fig. 2:**
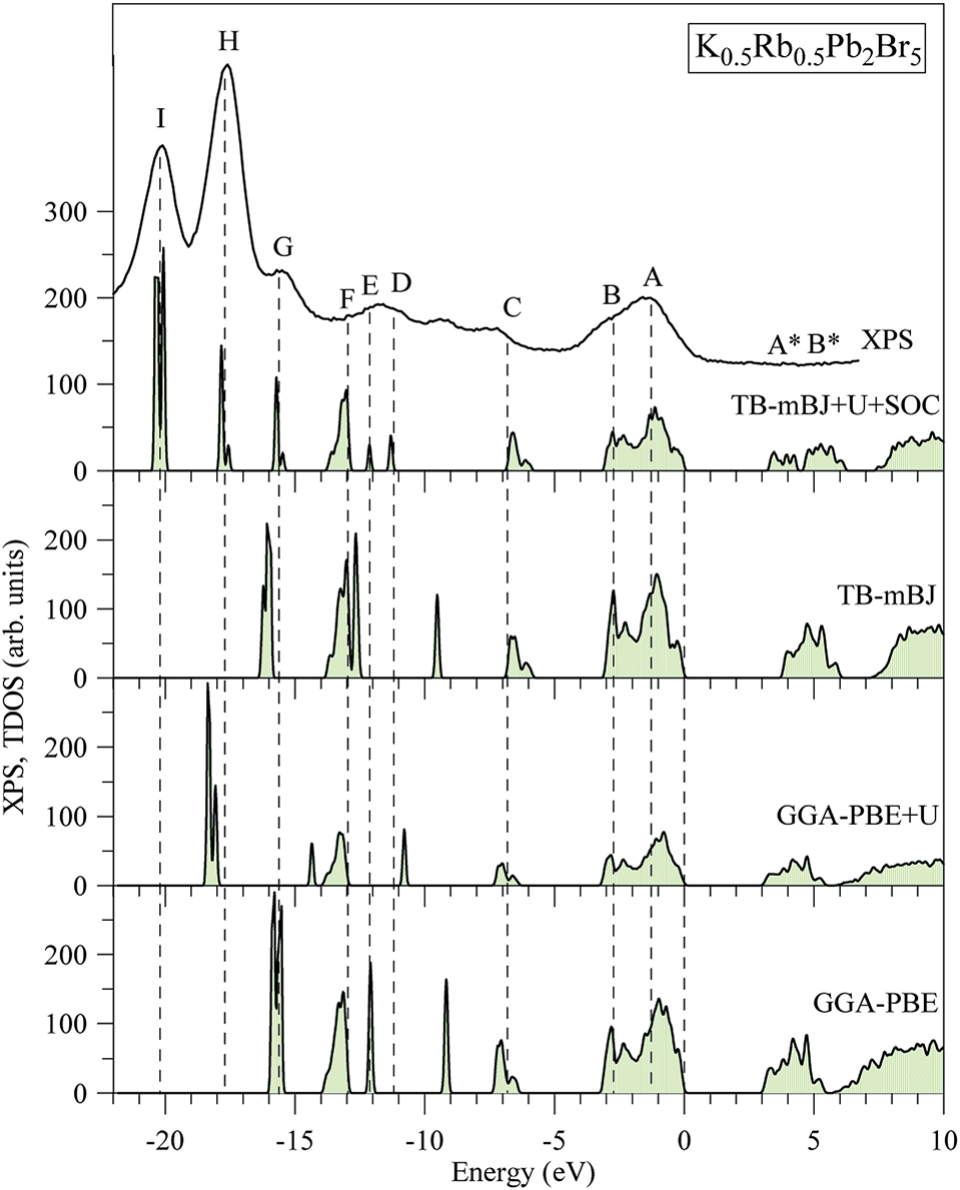
Total DOS of K_0.5_Rb_0.5_Pb_2_Br_5_ computed by employing the GGA-PBE, GGA-PBE + U, MBJ, and TB-mBJ + U + SOC techniques in comparison with the XPS spectrum of valence electrons of K_0.5_Rb_0.5_Pb_2_Br_5_ crystal.^22^ The Fermi level was shifted to zero energy level, while the XPS spectrum was shifted to the common energy scale of total DOS.

The electronic structures of K_0.5_Rb_0.5_Pb_2_Br_5_ obtained by GGA-PBE, GGA-PBE + U, TB-mBJ, and TB-mBJ + U + SOC, Fig. 2, are very different from each other. Thus, the valence band XPS spectrum of this compound^22^ is presented in Fig. 2 for finding the best correspondence of the theoretical results to the experiment. In general, the surface Fermi level of XPS spectra is higher than the valence band maximum.^54^ Therefore, the calculated total DOS was scaled by setting the top of valence band to zero energy level, and the XPS spectrum of valence electrons was shifted to ensure that the energy positions of the maxima on the curve of total density of states correspond to the positions of the main maxima (and “shoulders”) on the experimental X-ray photoelectron spectrum.

As it was reported in the work of Tarasova *et al.*,^22^ the band gap of KPb_2_Br_5_ is about 3.46 eV, while the band gap of RbPb_2_Br_5_ is 3.42 eV or 3.47 eV depending on the polarization. Because the band gap of K_0.5_Rb_0.5_Pb_2_Br_5_ is currently not available in published papers, it is considered to be some value between 3.42 and 3.47 eV. Based on this estimation, the GGA-PBE band gap is significantly smaller than the experimental values.^22^ It is widely accepted that GGA-PBE methods cause an over-delocalization of occupied states shifting the valence band to higher energy level, so the band gap is reduced. To compensate this effect, the Hubbard U parameter was introduced to GGA-PBE + U method. As a result, the valence band (VB) lower part is shifted to lower energy level by about 2 eV, as shown in Fig. 2. At the same time, the VB upper part is slightly moved, so the band gap is improved by only 0.012 eV. Taking into account the kinetic energy, and the Coulomb potential induced by the exchange hole in the TB-mBJ method,^55^ the K_0.5_Rb_0.5_Pb_2_Br_5_ band gap is increased by 0.758 eV in comparison with the GGA-PBE band gap. However, the electronic structure of K_0.5_Rb_0.5_Pb_2_Br_5_ is comparable with the XPS spectrum only when both U and SOC parameters were included in the TB-mBJ + U + SOC method. The TB-mBJ + U + SOC band gap is about 0.1 eV smaller than the smallest band gap of RbPb_2_Br_5_, however the separation of Pb-5d_3/2_ and Pb-5d_5/2_ is observed, and most of the main peaks (and shoulders) of the XPS spectrum are well reproduced. Since the TB-mBJ + U + SOC method reproduces the reliable electronic structure of K_0.5_Rb_0.5_Pb_2_Br_5_ in comparison with experimental data, it was used for studying further properties of this compound.

**Fig. 3:**
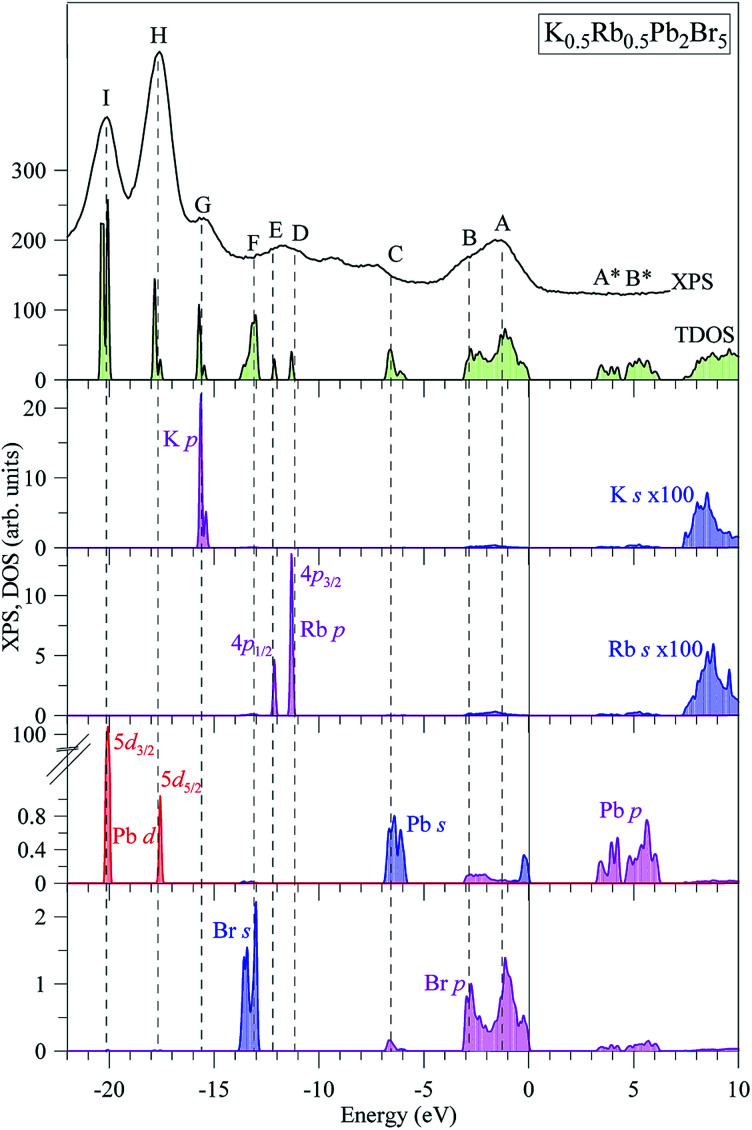
Total DOS and main partial densities of K_0.5_Rb_0.5_Pb_2_Br_5_ (computed by employing TB-mBJ + U + SOC method) in comparison with the XPS spectrum of valence electrons of K_0.5_Rb_0.5_Pb_2_Br_5_ crystal.^22^ The Fermi level was shifted to zero energy level, while the XPS spectrum was shifted to the common energy scale of total DOS.

The partial DOS curves of K_0.5_Rb_0.5_Pb_2_Br_5_ in Fig. 3 show that the Pb-5d_3/2_ and Pb-5d_5/2_ sub-states are located in the vicinities of −20 eV and −18 eV, respectively. The middle part of the VB is mainly formed by K-3p, Rb-4p, Br-4s states, in which the separation of Rb-4p_3/2_ and Rb-4p_1/2_ was also observed. The hybridization of Br-p and Pb-s/p states results in significant states contributions at −6.5 eV, and also near the valence band maximum (VBM). This reveals a major covalent part in the Br–Pb bonding. Contributions of the electronic states associated with potassium and rubidium are minor in the upper VB range as Fig. 3 demonstrates, however the present calculations predict some contributions of K-4s and Rb-5s states in the energy region between peculiarities A and B of the XPS spectrum of valence electrons of K_0.5_Rb_0.5_Pb_2_Br_5_. The conduction band minimum (CBM) is dominated by Pb-p and Br-p states, the upper parts are formed by Rb-s and K-s states.

**Fig. 4:**
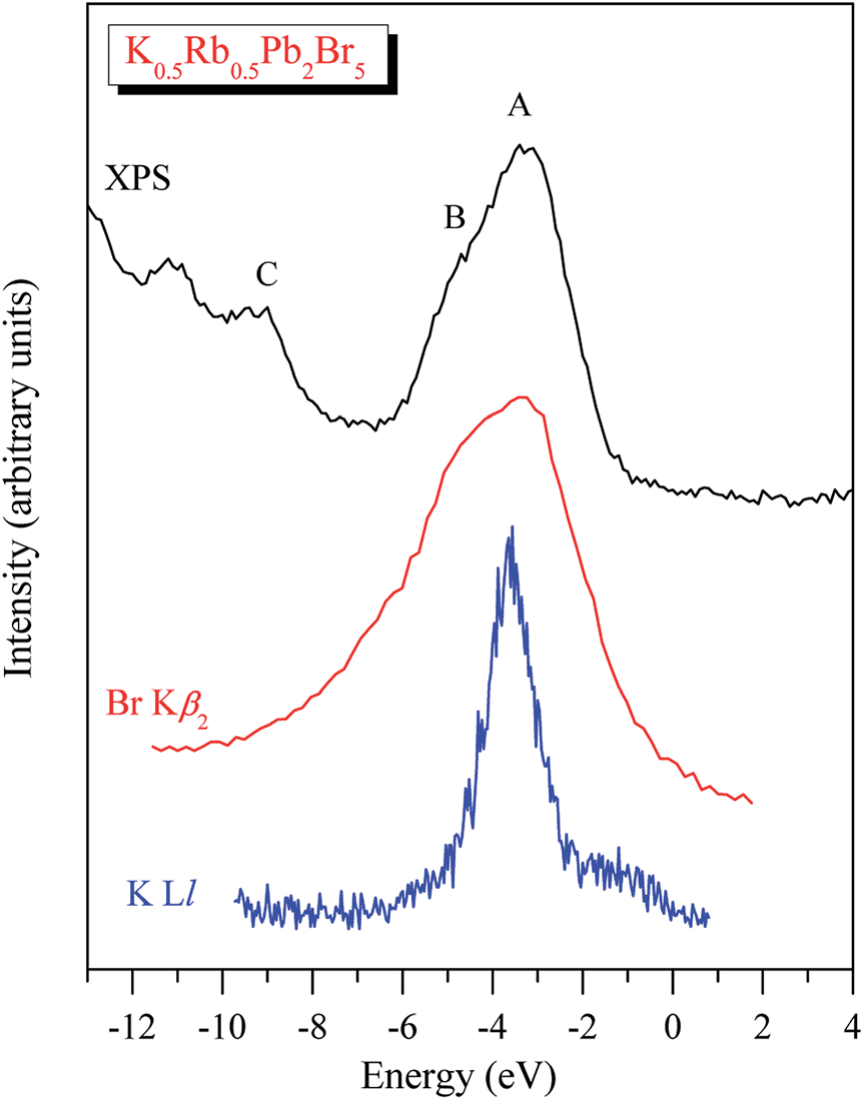
XPS spectrum of valence electrons of K_0.5_Rb_0.5_Pb_2_Br_5_ matched on a general energy scale with XES K Ll and Br Kβ_2_ spectra of this crystal.

In spite of the fact that contributions of K-4s states are minor in the VB range of K_0.5_Rb_0.5_Pb_2_Br_5_ as the data of the TB-mBJ + U + SOC calculations predict (Fig. 3), we were able to measure the energy distribution of these states experimentally. Results of comparison of the XES K Ll spectrum presenting energy distribution of K-4s states together with the Br Kβ_2_ spectrum bringing information on Br-4p electronic states, the main contributors to the valence band of K_0.5_Rb_0.5_Pb_2_Br_5_ in accordance with the present DFT calculations (Fig. 3), as well as the XPS spectrum of valence electrons of the bromide under discussion are presented in Fig. 4. Such a comparison of the XES and XPS spectra allows for statement that, the present experimental data yield that the maximum of the Br Kβ_2_ band is positioned in the vicinity of the maximum A of the XPS spectrum and reveals a significant input of Br-4p states in the vicinity of the peculiarity B, being in excellent agreement with partial Br-4p DOS curve shown in Fig. 3. Similar energy distribution of Br-4p states was found earlier to be characteristic of Pb- and Br-bearing counterparts APb_2_Br_5_ (A = K, Rb, Tl).^46,47,56^ Furthermore, the maximum of the K Ll band is located in the energy region between features A and B of the XPS spectrum (provided that a general energy scale is used for comparison of XES and XPS spectra), again agreeing with the TB-mBJ + U + SOC calculations of K_0.5_Rb_0.5_Pb_2_Br_5_. Therefore, the present TB-mBJ + U + SOC calculations with respect to main peculiarities of contributions of Br-4p and K-4s electronic states in the K_0.5_Rb_0.5_Pb_2_Br_5_ VB are fairly supported by XES and XPS experiments as comparison of Fig. 3 and 4 indicates. Certainly, some contributions of valence Pb-s and Pb-p states should come in the valence band of K_0.5_Rb_0.5_Pb_2_Br_5_ as visible from Fig. 3. Available database literature on X-ray emission lines and bands of chemical elements^57–60^ brings no information on the existence of XES spectra representing energy distribution of valence Pb-s and Pb-p states. Since lead is a heavy element, its outer (valence) states X-ray emission spectra should involve transitions of electrons in the VB levels (P orbitals) to vacancies in nearby inner core-levels (N or O orbitals) and such transitions should correspond to photons in ultra-soft X-ray range as it is reported to be characteristic of 5d-metal compounds.^60–63^ Probably, possibilities of synchrotron radiation should be used as it is proved to be sufficient in the case of compounds based on 5d-metals.^63,64^ This problem looks worth to be a task of special research because available experimental ability does not give us possibility to check experimentally the energy distribution of those states in the case of studying the valence band occupation in the bromide under discussion. Furthermore, the present DFT calculations yield that Rb-5s states should give minor contributions to the K_0.5_Rb_0.5_Pb_2_Br_5_ VB in the same energy region as K-4s electronic states bring. To probe Rb-5s states, X-ray emission spectrum should involve electron transitions from the O_I_ energy level to the inner M_II,III_ or N_II,III_ levels.^63^ However, the existence of such XES spectra has not been reported yet.^60,63,65^ Therefore, we leave this task for future research.

**Fig. 5:**
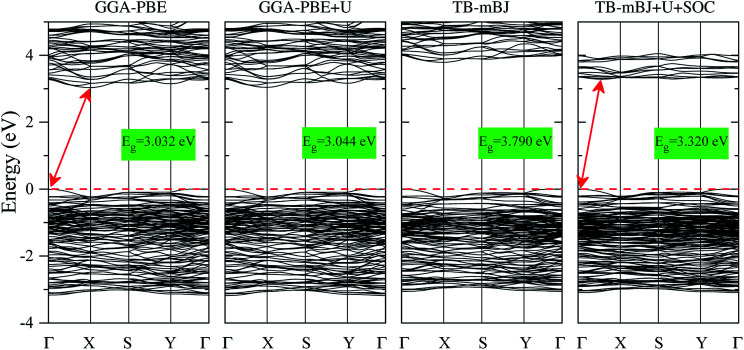
Band structures of K_0.5_Rb_0.5_Pb_2_Br_5_ calculated by GGA-PBE, TB-mBJ, GGA-PBE + U, and TB-mBJ + U + SOC techniques (the Fermi level is set to be at zero).

The band structures shown in Fig. 5 signify that K_0.5_Rb_0.5_Pb_2_Br_5_ is an indirect semiconductor with the VBM positioned at Γ-point and the CBM located near X-point. So, it requires less phonon for the momentum conversion in the electron–hole recombination process. While the Hubbard U parameter causes the minor effect on the band structure obtained by GGA-PBE and GGA-PBE + U methods, the exchange hole effect included in the TB-mBJ method induces a significant rise of unoccupied states widening the band gap. When introducing both U and SOC parameters in the TB-mBJ + U + SOC method, the CBM is slightly relocated from X-point and the unoccupied states in the conduction band are remarkably shifted down to lower energy levels which narrow band gap of K_0.5_Rb_0.5_Pb_2_Br_5_ to 3.320 eV.

### 4.2 Optical properties of K_0.5_Rb_0.5_Pb_2_Br_5_

The optical properties of K_0.5_Rb_0.5_Pb_2_Br_5_ can be obtained based on TB-mBJ + U + SOC calculations of the wave function |*knp*〉 and eigenvalue energy *E*_*kn*_ in a unit-cell volume *V* with crystal momentum *k* and spin *σ*. Then, the real *ε*_1_(*ω*) and imaginary *ε*_2_(*ω*) parts of the dielectric function *ε*(*ω*) can be calculated following Kramers–Kronig relations:^66^1
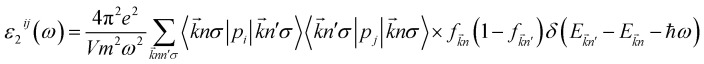
and2
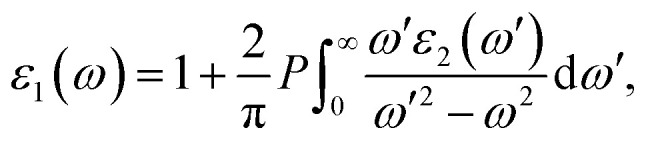
where *f*_*kn*_, and *P* are Fermi distribution function, and Cauchy principal value; *m*, *e*, *ω* and *p* are the electron mass and charge, and the momentum operator, respectively. The obtained value matrix of dielectric functions *ε*_1_^*ij*^(*ω*) and *ε*_2_^*ij*^(*ω*) can be used to calculate the extinction coefficient *n*(*ω*) and the refractive index *k*(*ω*) as follows:^67^3
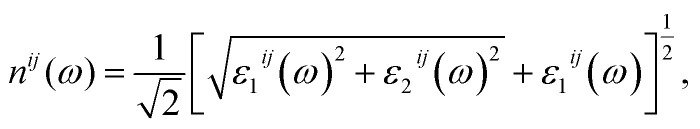
4
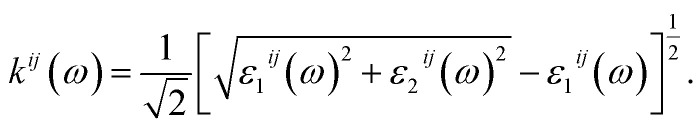


Finally, the adsorption coefficient *α*(*ω*), optical reflectance *R*(*ω*), and the energy loss spectrum *L*(*ω*) were calculated:5
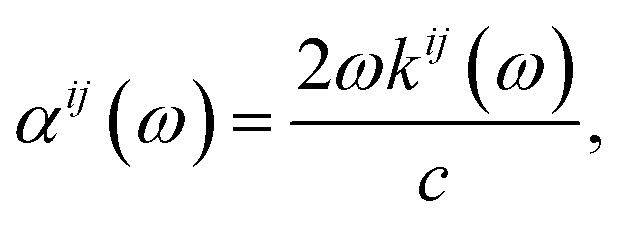
6
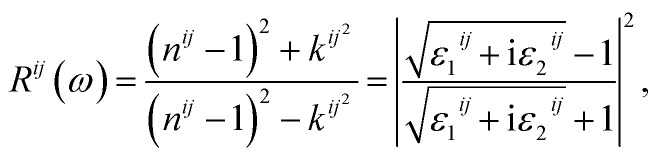
7
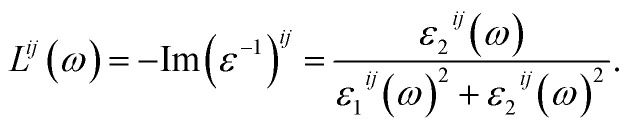


**Fig. 6:**
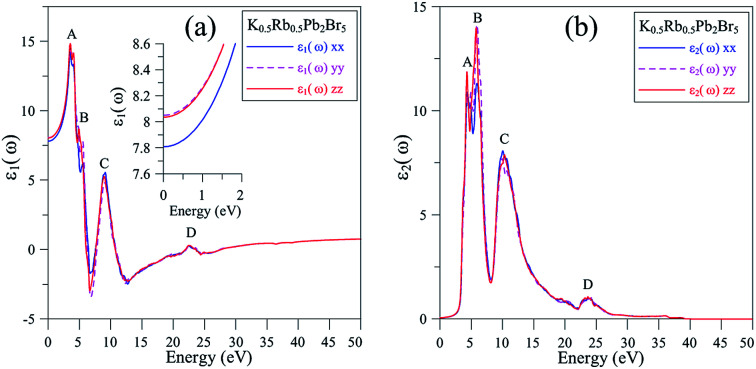
(a) Real and (b) imaginary components of dielectric function *ε*(*ω*) of K_0.5_Rb_0.5_Pb_2_Br_5_ (calculated with TB-mBJ + U + SOC).

It is shown in Fig. 6(a) that the polarization of *ε*_1_(*ω*) function is only recognizable at the extrema of its spectrum. The reflectivity of K_0.5_Rb_0.5_Pb_2_Br_5_ is high at about 4 eV, where *ε*_1_(*ω*) functions reach their maximum values. The polarization is also remarkable in the vicinity of 5 eV, where the permittivity along the *y*/*z*-directions is higher than along the *x*-direction. In the energy range of 5–7 eV, there is sharp decrease of *ε*_1_(*ω*) functions to negative values resulting in the damping of electromagnetic (EM) wave and a reverse polarization where the intensity of *ε*_1_^*xx*^(*ω*) is higher than the ones of *ε*_1_^*yy*^(*ω*) and *ε*_1_^*zz*^(*ω*). For energy level higher than 7 eV, the polarization becomes minor, and the *ε*_1_(*ω*) functions sharply increase to the third highest peak followed by weak response to the incident EM wave with little fluctuation.

The inter-band transition of electron in K_0.5_Rb_0.5_Pb_2_Br_5_ is featured by the *ε*_2_(*ω*) spectrum in Fig. 6(b), where remarkable phonon absorption is recognized at the energy level of about 5 eV corresponding to the first *ε*_2_(*ω*) extremum. The polarization of *ε*_2_(*ω*) is observed in the energy range of 5–6 eV, where *ε*_2_^*yy*^(*ω*) and *ε*_2_^*zz*^(*ω*) are higher than *ε*_2_^*xx*^(*ω*). At 6 eV, *ε*_2_(*ω*) functions reach the highest value with strongest polarization, before a sharp decrease to the negative concave down point at 8 eV. For energy level higher than 8 eV, the *ε*_2_(*ω*) functions rise up again to the third highest peak at 11 eV where the reverse polarization occurs. The inter-band transition of electron is expected to be at highest rate in the energy range of 4–7 eV. This range can be narrowed by doping with rare-earth ions, as in the cases of rare-earth doped KPb_2_Br_5_, and RbPb_2_Br_5_,^13,68,69^ making the rare-earth doped K_0.5_Rb_0.5_Pb_2_Br_5_ be one of the most promising laser materials operating in the mid-infrared energy range. The second highest extremum of *ε*_2_(*ω*) is found in the vicinity of 10–12 eV. This signifies a remarkably high inter-band transition between valence core levels near −6.5 eV to the conduction band, which is an advantage for core valence luminescence induced by impurities in such halides with large band gap like K_0.5_Rb_0.5_Pb_2_Br_5_.^70–73^

**Fig. 7:**
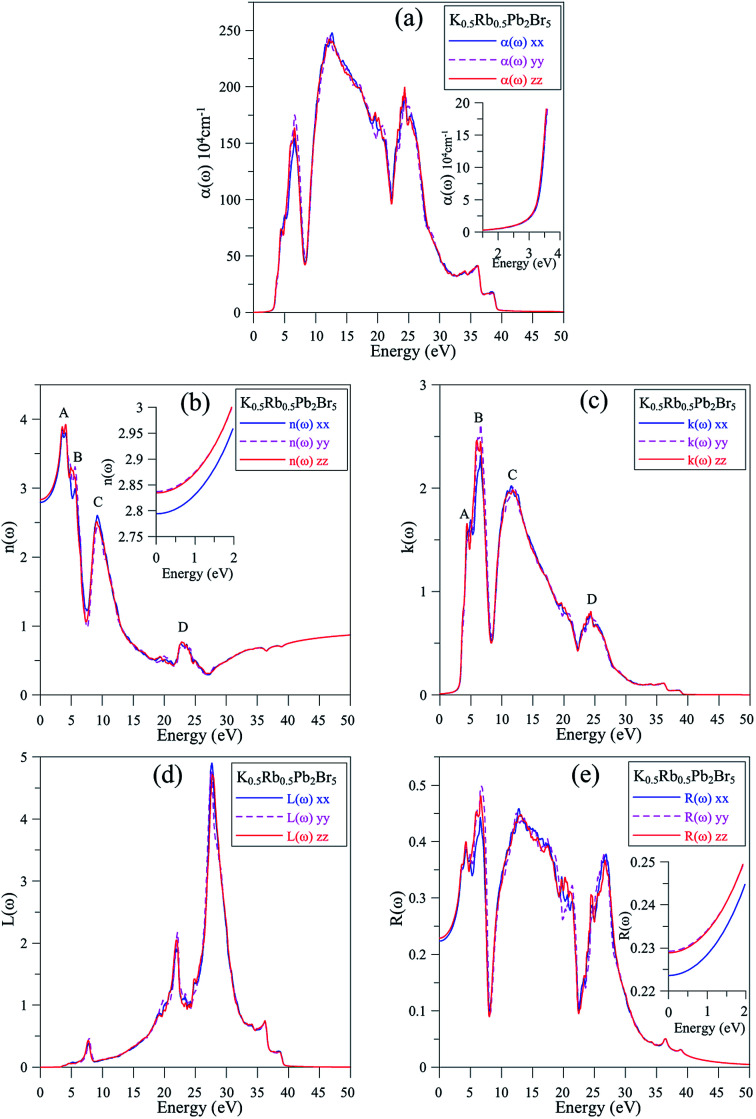
(a) Absorption coefficient *α*(*ω*), (b) refractive index *n*(*ω*), (c) extinction coefficient *k*(*ω*), (d) electron energy-loss spectrum *L*(*ω*) and (e) optical reflectivity *R*(*ω*) of K_0.5_Rb_0.5_Pb_2_Br_5_ (calculated by TB-mBJ + U + SOC).

In Fig. 7(a), the absorption edge of K_0.5_Rb_0.5_Pb_2_Br_5_ is positioned near 3.5 eV indicating the transparency of this compound for EM wave with wave-length longer than 354 nm. For the energy range of 5–25 eV, the absorption coefficient *α*(*ω*) can reach the order of magnitude of 10^6^ cm^−1^ at 7 eV, 12 eV, and 24 eV where significant polarization is also observed. In the vicinity of 7 eV, the *α*^*yy*^(*ω*) and *α*^*zz*^(*ω*) functions are higher than *α*^*xx*^(*ω*), then the first reverse polarization occurs at the concave down point at 8 eV, and the polarization changes again at concave down point at 22 eV. The extremum of *α*(*ω*) at a very high energy level of 24 eV can be explained by the inter-band transition between the Pb-5d_3/2_ and Pb-5d_5/2_ sub-states and the unoccupied states in the conduction band.

The reflectivity *R*(*ω*) of K_0.5_Rb_0.5_Pb_2_Br_5_ plotted in Fig. 7(e) is characterized by the rate of 40–50%. The reflectivity *R*(*ω*) is anisotropic in the vicinities of 5 eV, 13 eV, and 27 eV. The reverse polarization is observed at concave down points at 8 eV, and 22 eV. For the energy level lower than 3.5 eV, the absorption coefficient *α*(*ω*) is almost zero, so the reflectivity may origin from the oscillations of the polarization of atoms. At the energy level ranging from 4 eV to 27 eV, the reflectivity *R*(*ω*) increases significantly together with the increase of *α*(*ω*), but not at the same rate. The refractive index *n*(*ω*), the extinction coefficient *k*(*ω*), and the energy loss *L*(*ω*) were calculated and presented in Fig. 7(b)–(d).

Fig. 7(b) and (c) show good relationship of shapes between *n*(*ω*) and *k*(*ω*) with *ε*_1_(*ω*) and *ε*_2_(*ω*), respectively. The *n*(*ω*), spectrum starts at static value of about 2.8, with the difference due to polarization of about 0.05, indicating the existence of birefringence in K_0.5_Rb_0.5_Pb_2_Br_5_. The *n*(*ω*) spectrum increases to the highest peaks of 3.8–3.9 at energy range of 3–5 eV. This causes the largest change in the velocity of EM wave with wave-length of 348–413 nm in the K_0.5_Rb_0.5_Pb_2_Br_5_ compound. The reverse polarization is found at the concave down point at 5.5 eV, then, at 10 eV *n*(*ω*), it reaches its highest peak where *n*^*xx*^(*ω*) is higher than *n*^*yy*^(*ω*) and *n*^*zz*^(*ω*). The extinction coefficient *k*(*ω*) is remarkable in the energy range of 5–14 eV with the highest peak at 6 eV, the attenuation decreases gradually for energies higher 15 eV. The loss energy *L*(*ω*) shown in Fig. 7(d) is very small for energy level lower than 28 eV which is opposite to behavior of the absorption coefficient *α*(*ω*) in Fig. 7(a), this signifies a minor thermalization owing to the sub-band transition of the electron in the conduction band. However, in the vicinity of 28 eV both *L*(*ω*) and *α*(*ω*) reach their high peaks because of strong interaction of atoms and photons of these energies.

## 5 Conclusion

The electronic structure and optical properties of K_0.5_Rb_0.5_Pb_2_Br_5_ semiconductor were studied by applying the first-principle calculation within Kohn–Sham framework. The GGA-PBE and TB-mBJ methods with and without Hubbard U and SOC parameters were employed to investigate the electronic structure of K_0.5_Rb_0.5_Pb_2_Br_5_ under the effects of kinetic energy, Coulomb potential induced by exchange hole, and spin-orbital interaction. It was found that the TB-mBJ + U + SOC method well reproduces electronic structure of K_0.5_Rb_0.5_Pb_2_Br_5_ in comparison with XPS spectrum.

The band gap of K_0.5_Rb_0.5_Pb_2_Br_5_ was determined for the first time to be indirect, and the theoretically predicted value is of 3.32 eV. The Pb-5d_3/2_ and Pb-5d_5/2_ sub-states are located in vicinities of −20 eV and −18 eV, respectively. The middle part of the valence band is mainly formed by K-3p, Rb-4p, Br-4s states, in which the separation of Rb-4p_3/2_ and Rb-4p_1/2_ was also observed. The valence band maximum (VBM) is mostly composed of hybridization of Br-p and Pb-s/p states. While the conduction band minimum (CBM) is dominated by Pb-p and Br-p states, the upper part is constructed by Rb-s and K-s states. The TB-mBJ + U + SOC calculations concerning main peculiarities of contributions of Br-4p and K-4s electronic states in the K_0.5_Rb_0.5_Pb_2_Br_5_ VB are fairly supported by XES and XPS experiments.

The reflectivity of K_0.5_Rb_0.5_Pb_2_Br_5_ is high for photons with energies of about 4 eV. In the energy range from 5 eV to 7 eV, the *ε*_1_(*ω*) functions undergo a sharp decrease to negative values resulting in the damping of electromagnetic wave and a reverse polarization where the intensity of *ε*_1_^*xx*^(*ω*) is higher than the ones of *ε*_1_^*yy*^(*ω*) and *ε*_1_^*zz*^(*ω*). For energy level higher than 7 eV, K_0.5_Rb_0.5_Pb_2_Br_5_ weakly responds to the incident electromagnetic wave. The phonon absorption is remarkable at 5 eV. The polarization of *ε*_2_(*ω*) is observed in the energy range from 5 eV to 6 eV, where *ε*_2_^*yy*^(*ω*) and *ε*_2_^*zz*^(*ω*) are higher than *ε*_2_^*xx*^(*ω*). At 6 eV, *ε*_2_(*ω*) functions reach the highest value with strongest polarization. The reverse polarization occurs at 8 eV. The inter-band transition of electron is expected to be at highest rate in the energy range from 4 eV to 7 eV. This range can be narrowed by doping with rare-earth ions, making the rare-earth doped K_0.5_Rb_0.5_Pb_2_Br_5_ be one of the most promising laser materials operating in the mid-infrared energy range. The second highest extremum of *ε*_2_(*ω*) is found in the vicinity of 10–12 eV. This signifies a remarkably high inter-band transition between valence core levels near −6.5 eV to the conduction band, which is an advantage for core valence luminescence induced by impurities.

The K_0.5_Rb_0.5_Pb_2_Br_5_ compound is transparent for electromagnetic wave with wave-length longer than 354 nm. For the energy range from 5 eV to 25 eV, the absorption coefficient *α*(*ω*) can reach the order of magnitude of 10^6^ cm^−1^ with three main extrema at 7 eV, 12 eV, and 24 eV where significant polarization is also observed. The reverse polarization occurs at the concave down points at 8 eV, and 22 eV. K_0.5_Rb_0.5_Pb_2_Br_5_ reveals significant birefringence at energy levels in the vicinity of 5 eV. Electromagnetic waves with wave-length in the interval from 348 nm to 413 nm undergo a remarkable change of velocity in the K_0.5_Rb_0.5_Pb_2_Br_5_ compound. The extinction coefficient *k*(*ω*) is remarkable in the energy range from 5 eV to 14 eV with the highest peak at 6 eV, the attenuation decreases gradually for energies higher 15 eV. For energy levels lower than 28 eV, minor thermalization may happen owing to the sub-band transition of the electron in the conduction band. However, in the vicinity of 28 eV both *L*(*ω*) and *α*(*ω*) reach their high peaks because of the strong interaction of atoms and photons of these energies. Generally, the fact that the polarization of optical properties occurs at the extrema in the optical spectra. Despite that fact that the polarization is minor in most of the cases, it is necessary to be taken into account when using K_0.5_Rb_0.5_Pb_2_Br_5_ as an active host for laser materials.

## Conflicts of interest

There are no conflicts to declare.

## Supplementary Material
